# Reconfigurable Magnetotransport in MnBi_2_Te_4_ via Gate and Magnetic Field Tuning

**DOI:** 10.1002/adma.202510734

**Published:** 2025-09-26

**Authors:** Yuang Jie, Xiaofan Cai, Yijie Lin, Kenji Watanabe, Takashi Taniguchi, Jiaqiang Yan, Dmitry Ovchinnikov, Ahmet Avsar

**Affiliations:** ^1^ Department of Materials Science and Engineering National University of Singapore Singapore 117575 Singapore; ^2^ Research Center for Electronic and Optical Materials National Institute for Materials Science 1‐1 Namiki Tsukuba 305‐0044 Japan; ^3^ Research Center for Materials Nanoarchitectonics National Institute for Materials Science 1‐1 Namiki Tsukuba 305‐0044 Japan; ^4^ Materials Science and Technology Division Oak Ridge National Laboratory Oak Ridge TN 37831 USA; ^5^ Department of Physics and Astronomy University of Kansas Lawrence KS 66045 USA; ^6^ Department of Physics National University of Singapore Singapore 117542 Singapore; ^7^ Centre for Advanced 2D Materials National University of Singapore Singapore 117546 Singapore

**Keywords:** gate tuning, in‐plane magnetic field, magnetic anisotropy, magnetotransport, MnBi_2_Te_4_

## Abstract

The intrinsic magnetic topological insulator MnBi_2_Te_4_ is a promising platform for exploring quantum phases with nontrivial band topology and for enabling electrical control over coupled magnetic and electronic phase transitions. In‐plane magnetic fields, in particular, offer a distinct means of tuning these properties by strengthening quantized Hall effects, enhancing surface energy gaps, and driving spin reorientation transitions. However, a systematic understanding of how such fields affect magnetotransport is limited. Here, the magnetotransport behavior of few‐layer MnBi_2_Te_4_ as a function of gate voltage, temperature, and magnetic field angle, with a primary focus on in‐plane field effects, are investigated. A gate‐tunable crossover in magnetoresistance is observed from positive to negative values under in‐plane magnetic fields as the gate voltage is swept below the charge neutrality point at temperatures below the Néel temperature. The in‐plane field drives a transition from the antiferromagnetic ground state to a ferromagnetic configuration with spins aligned in‐plane, while simultaneously altering the electronic structure, as revealed by gate‐dependent transport features. The angle‐dependent measurements reveal strongly gate‐tunable magnetotransport anisotropy. These results establish in‐plane magnetic fields as an effective tuning parameter for modulating spin and charge transport in MnBi_2_Te_4_, advancing prospects for reconfigurable spintronic and topological devices.

## Graphical abstract

Gate‐tunable magnetoresistance sign reversal under in‐plane magnetic fields is observed in an ultrathin MnBi_2_Te_4_ device, arising from spin reorientation and distinct scattering mechanisms. Moreover, angular‐dependent measurements reveal clear gate‐controlled magnetotransport anisotropy, underscoring the strong coupling between gating, spin configuration, and transport behavior in MnBi_2_Te_4_.

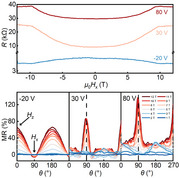

## 1 Introduction

MnBi_2_Te_4_ (MBT), a layered van der Waals antiferromagnet, has emerged as a compelling platform for exploring the interplay between magnetism, band topology, and electronic transport phenomena.^[^ ^1^, ^2^, ^3^, ^4^, ^5^, ^6^, ^7^ ^]^ Below its Néel temperature (*T*_N_ ∼ 25 K in bulk^[^ ^1^ ^]^), MBT exhibits A‐type antiferromagnetic (AFM) order, with ferromagnetically aligned spins within each septuple layer and AFM coupling between adjacent layers along the crystallographic *c*‐axis.^[^ ^2^, ^5^ ^]^ When subjected to an out‐of‐plane magnetic field, the spin configuration undergoes a series of well‐defined transitions—from the AFM ground state to an intermediate spin‐flop state, and eventually into a fully spin‐polarized ferromagnetic (FM) state.^[^ ^8^ ^]^ These magnetic transitions manifest in magnetotransport signatures that are highly sensitive to carrier density, enabling gate‐tunable control over the spin order and the associated topological band structure.^[^ ^6^, ^9^, ^10^, ^11^, ^12^ ^]^

While the response of bulk^[^ ^11^, ^13^ ^]^ and few‐layer^[^ ^1^, ^8^, ^14^ ^]^ MBT to out‐of‐plane field is now well established; in‐plane magnetic fields present a fundamentally different and increasingly relevant tuning axis. In‐plane fields can strongly influence the magnetic state by inducing non‐collinear spin textures, which in turn reshape the Berry curvature landscape, offering pathways to tuning correlated and topological transport phenomena.^[^ ^15^, ^16^, ^17^, ^18^, ^19^ ^]^ Recent studies have demonstrated that modest in‐plane fields can enhance the robustness of quantized Hall states in MBT by stabilizing magnetic order and reducing dissipation.^[^ ^20^ ^]^ Moreover, angle‐dependent transport measurements have shown that in‐plane fields can drive topological phase transitions from a Chern insulator to a trivial magnetic insulator by gapping the Dirac surface states through spin realignment.^[^ ^21^ ^]^ These field‐driven spin reconfigurations, particularly at the surface, enhance the out‐of‐plane magnetization component and increase the surface energy gap, promoting topological robustness while simultaneously allowing for anisotropic backscattering and large magnetoresistance (MR) effects. These findings highlight the growing relevance of in‐plane field control, yet a comprehensive understanding of how MBT's magnetotransport behavior evolves under in‐plane fields, particularly in relation to gate voltage and temperature, remains largely absent.

In this work, we perform a comprehensive study of magnetotransport in few‐layer MBT devices subjected to in‐plane magnetic fields, focusing on the evolution of resistance, critical fields, and magnetic anisotropy across a wide gate voltage and temperature range. A pronounced gate‐tunable crossover in MR, from negative to positive values, is observed below *T*_N_ as the Fermi level is tuned. The in‐plane magnetic field not only drives a spin reorientation from the AFM ground state to the FM configuration, but also modulates the underlying band structure, as evidenced by gate‐dependent shifts in the position of the charge neutrality point (CNP). Angle‐dependent MR measurements reveal a strong gate modulation of magnetic anisotropy, while temperature‐dependent studies of MR evolution elucidate the dominant transport mechanisms in the high‐field regime and their connection to long‐range magnetic order. These results provide detailed insight into the coupled spin and electronic structure evolution of MBT under in‐plane fields and establish a foundation for designing field‐ and gate‐tunable spintronic functionalities in layered antiferromagnets.

## 2 Results and Discussion

### 2.1 Device Structure and Basic Characterization

**Figure 1:**
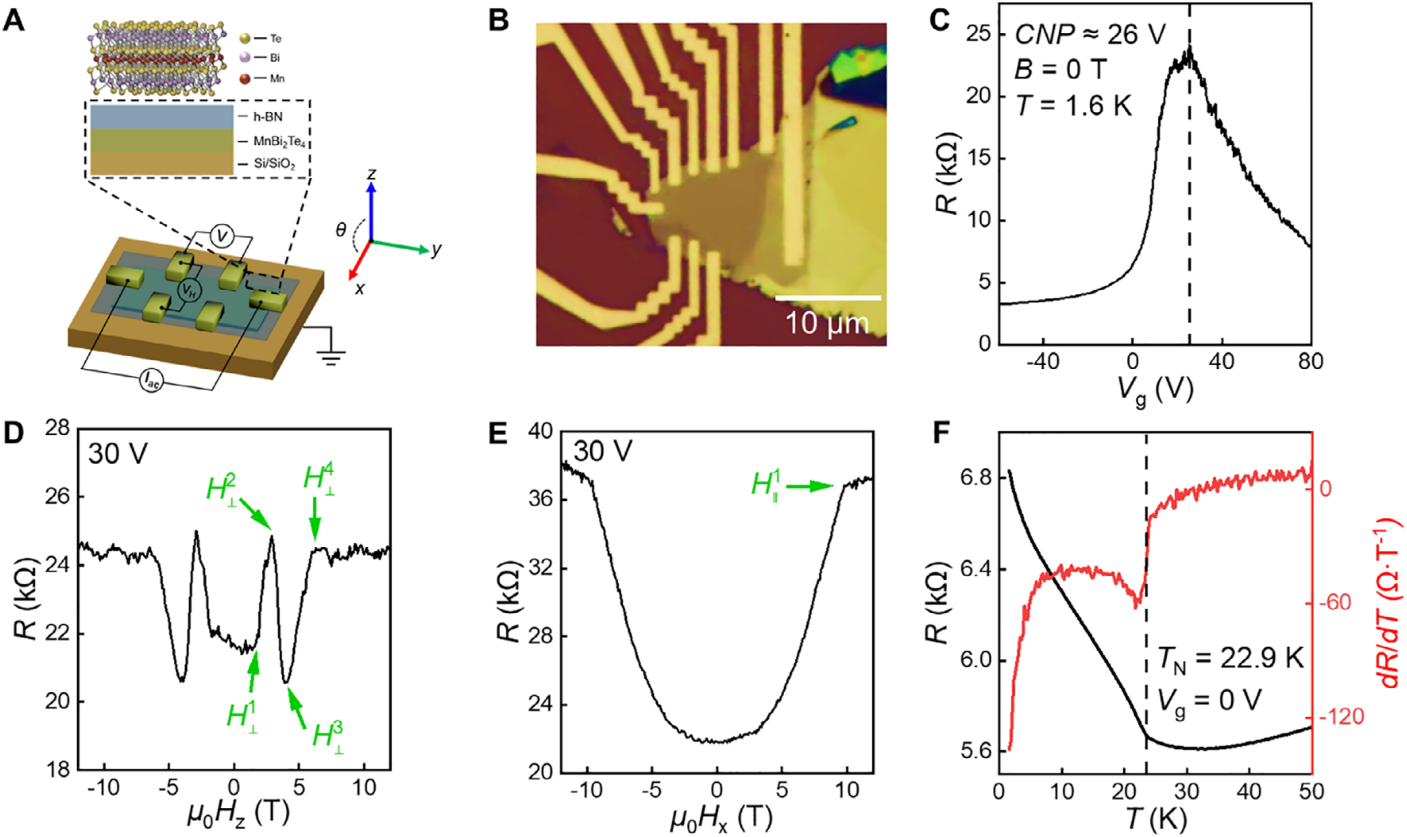
Device structure and basic magnetotransport characterization of MBT. A) Schematic illustration of the MnBi_2_Te_4_ crystal structure and device architecture. The device consists of an h‐BN/MBT/SiO_2_/Si heterostructure with patterned electrical contacts. Coordinate definition: current flows along the y‐axis; magnetic field is applied either along the z‐axis (out‐of‐plane), x‐axis (in‐plane) or at intermediate angles within the x–z plane. B) Optical microscope image of Device 3. C) Gate‐dependent resistance for Device 3 measured at 1.6 K and zero magnetic field. The dashed line marks the position of the CNP, located near 26 V. D) *R*–*μ*_0_ *H* curve under out‐of‐plane magnetic field near CNP, showing four characteristic fields $H_{⊥}^{1}$, $H_{⊥}^{2}$, $H_{⊥}^{3}$ and $H_{⊥}^{4}$. E) *R*–*μ*_0_ *H* curve under in‐plane magnetic field near CNP, showing the characteristic field $H_{∥}^{1}$. F) Temperature dependent resistance measured at zero field and zero gate voltage. The dashed line indicates the respective Néel temperatures determined by the abrupt change in the *dR/dT* versus *T* curve.

**Figure** **1** provides an overview of the device architecture and representative magnetotransport characteristics of the MBT samples studied. High‐quality MBT crystals were mechanically exfoliated in a nitrogen‐filled glovebox to avoid air exposure. For long‐term stability and reliable device performance, the flakes were encapsulated with a 3–5 layer hexagonal boron nitride (h‐BN) sheet, which also serves as a tunneling‐transparent interface for efficient charge injection.^[^ ^22^ ^]^ As illustrated in Figure 1A, the resulting device consists of a few‐layer MBT flake supported on a SiO_2_/Si substrate. The measurement geometry is defined with current flowing along the y‐axis and magnetic fields applied either out‐of‐plane (along the z‐axis), in‐plane (along the x‐axis), or at intermediate angles within the x–z plane, as illustrated in Figure 1A. Samples were characterized through systematic magnetotransport measurements as a function of back gate voltage, magnetic field orientation, and temperature. Unless otherwise specified, all measurements shown were performed at *T*  =  1.6 K. In total, four devices were studied, all exhibiting consistent behavior; here we focus on a representative device with a flake thickness of 12.6 nm (9 layers) (see Figure S1, Supporting Information). We focus our measurements on the thinner region of the flake in Device 3. An optical image of this device is shown in Figure 1B. Additional four‐terminal and two‐terminal measurements conducted on other devices show consistent results, with closely matching behavior across devices, as illustrated in Figures S2–S4 (Supporting Information).

Figure 1C shows the two‐terminal resistance as a function of back gate voltage, measured at zero magnetic field. A pronounced resistance peak is observed near 26 V, corresponding to the CNP. Figure 1D shows the magnetotransport behavior of the device under an out‐of‐plane magnetic field near CNP. Four characteristic fields, $H_{⊥}^{1}$, $H_{⊥}^{2}$, $H_{⊥}^{3}$, and $H_{⊥}^{4}$, are clearly identified in the MR curve, corresponding to distinct magnetic transitions: $H_{⊥}^{1}$ marks the onset of the spin‐flop transition, below which MBT remains in AFM state. $H_{⊥}^{2}$ and $H_{⊥}^{3}$ correspond to the resistance peak and valley during the spin‐flop process. $H_{⊥}^{4}$ indicates the field at which the system fully transitions into the FM state. These features indicate the progressive evolution of the A‐type AFM structure of MBT into the FM state under increasing out‐of‐plane field strength.^[^ ^8^, ^23^ ^]^ To compare, Figure 1E displays the in‐plane MR response under otherwise identical conditions. A distinct transition is observed at $H_{∥}^{1}$, representing the critical field for reorienting spins from out‐of‐plane to fully in‐plane alignment, thereby completing the AFM‐to‐FM transformation.^[^ ^24^ ^]^ Finally, Figure 1F shows the temperature dependence of the resistance and its first‐order derivative at zero gate voltage and zero magnetic field. The *T*_N_ is determined from the inflection point in the derivative and is found to be 22.9 K (±0.4 K), consistent with prior reports on high‐quality, thin MBT crystals.^[^ ^1^ ^]^

### 2.2 In‐Plane Magneto‐Transport

**Figure 2:**
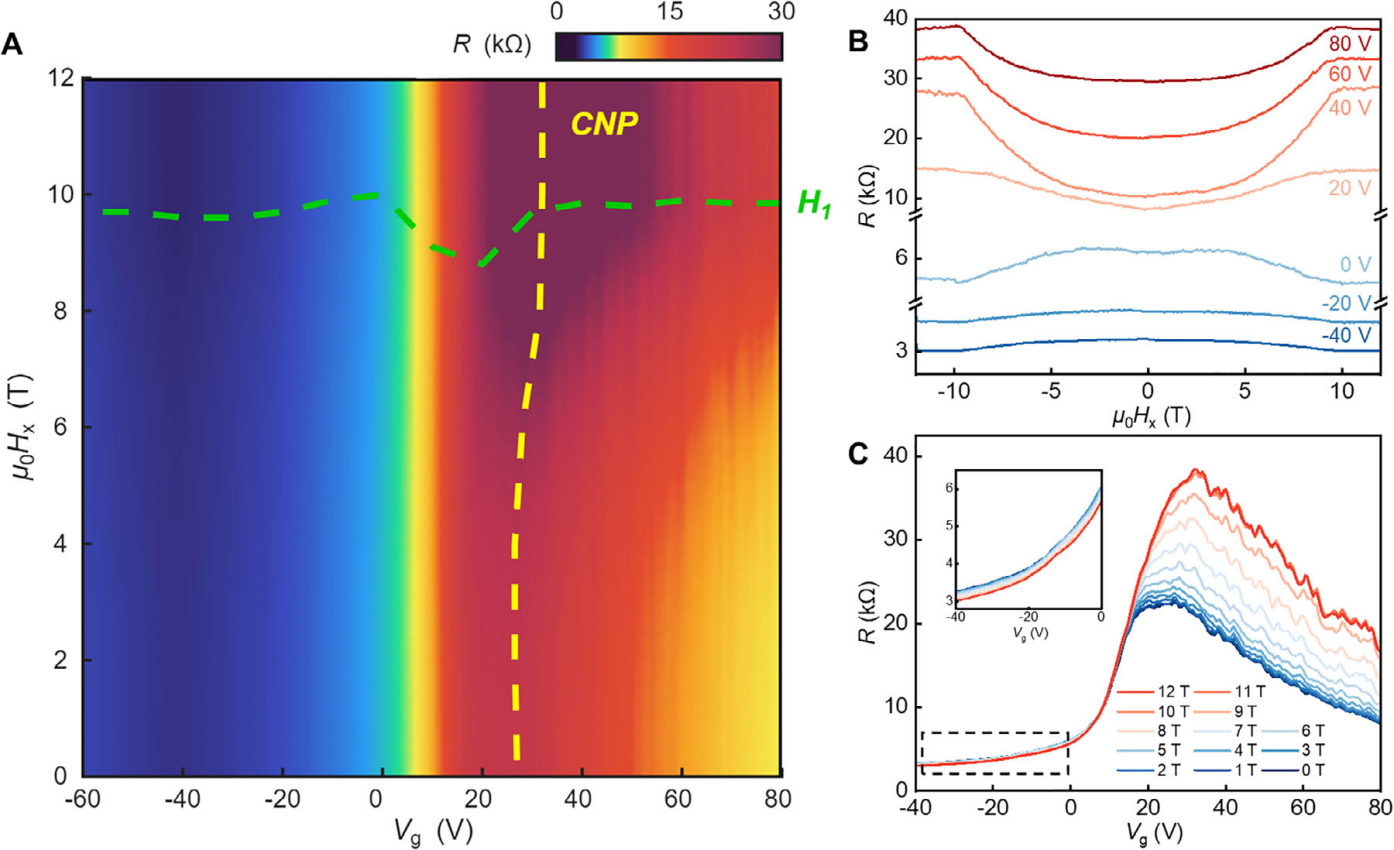
Gate‐dependent magnetotransport of MBT under in‐plane magnetic field at 1.6 K. A) Colormap of resistance as a function of in‐plane magnetic field and gate voltage, with the evolution of characteristic field $H_{∥}^{1}$ and CNP marked in green and yellow dashed lines, respectively. B) *R*–*B* curves under in‐plane magnetic field at different gate voltages, vertically offset for clarity. C) Two‐terminal resistance as a function of magnetic fields at different in‐plane magnetic fields. The inset highlights a magnified view of the region marked by the dashed rectangle.

To investigate the underlying tuning mechanisms and their correlation with the band structure, gate‐dependent magnetotransport measurements were performed under in‐plane magnetic fields. **Figure** **2**A presents a colormap of the longitudinal resistance as a function of in‐plane magnetic field and back gate voltage. The green and yellow dashed lines trace the evolution of the critical field $H_{∥}^{1}$, associated with the spin reorientation from the layered A‐type AFM ground state to the in‐plane polarized FM state, and the position of the CNP, respectively (see Figure S5, Supporting Information for details). These trends reveal that in‐plane fields enable continuous tuning of both spin configuration and electronic structure. Unlike the case under out‐of‐plane magnetic fields, no band crossing or abrupt topological transition is observed within the explored field and gate range.^[^ ^7^ ^]^ Horizontal and vertical line cuts from this colormap are presented in Figure 2B,C, respectively, and are discussed in the following sections.

Figure 2B shows the in‐plane magnetic field dependence of resistance at several fixed gate voltages. Both the shape and magnitude of the MR evolve systematically with gating. The critical field $H_{∥}^{1}$​, extracted from the derivative d*R*/d(*μ*_0_ *H*), decreases from 9.9 T (±0.1 T) at high carrier densities to 8.7 T (±0.1 T) near *V*_g_ = 20 V (±0.5 V) (see Figure S5, Supporting Information). For out‐of‐plane magnetic fields, it has been shown that the MR sign undergoes a reversal from positive to negative as the gate voltage tunes the system from hole to electron carriers.^[^ ^12^, ^14^ ^]^ By comparison, our measurements exhibit a distinct behavior. At large negative gate voltages, the MR, defined as *R*(12 T) – *R*(0 T), is negative and gradually weakens as the gate voltage increases. At *V*_g_ = 10 V (±0.5 V), the MR becomes nearly flat, showing minimal field dependence. At higher gate voltages, it turns positive and increases in magnitude. The gate‐dependent sign reversal in in‐plane MR likely reflects a crossover between distinct scattering regimes. For gate voltages below 10 V, where the Fermi level is expected to reside in the bulk valence band, magnetic fields could suppress spin fluctuations and reduce spin‐dependent scattering. This could cause the observed negative MR, similar to conventional ferromagnetic systems.^[^ ^25^, ^26^, ^27^ ^]^ As the gate voltage increases beyond 10 V, the Fermi level may enter the gapped surface state region, where in‐plane magnetic fields could distort the Dirac cone and weaken spin‐momentum locking.^[^ ^17^, ^18^, ^28^, ^29^, ^30^ ^]^ This distortion could enhance spin‐dependent scattering and contribute to a positive MR. Notably, the positive MR persists even at high positive gate voltages, suggesting that additional mechanisms such as subtle changes in band topology or spin texture may be at play. The same gate‐tunable crossover in MR polarity was also observed in three additional devices, as shown in Figures S2–S4 (Supporting Information), confirming the robustness of this behavior across samples. We also performed two‐terminal MR measurements using different electrode configurations along various in‐plane directions, as illustrated in Figure S6 (Supporting Information), all exhibited similar trends. Furthermore, theoretical studies have reported that the exchange interactions in MBT are nearly isotropic within the ab‐plane.^[^ ^31^ ^]^ Taken together, the consistency between our experimental observations and theoretical expectations suggests that MBT can be regarded as effectively isotropic in‐plane. Notably, even after the system enters the FM state at high fields, the resistance continues to rise without signs of saturation, indicating the presence of residual scattering or additional field‐dependent transport channels—an aspect further examined in the temperature‐dependent analysis.

Figure 2C presents *R*–*V*_g_ curves at a series of fixed in‐plane magnetic fields. The position of the CNP remains nearly unchanged up to *μ*_0_ *H* = 5 T. Beyond this field, the CNP shifts steadily from 23 V (±0.5 V) at 5 T to 32 V (±0.5 V) at 9 T, just below the critical field $H_{∥}^{1}$. Once the system enters the in‐plane FM regime, the CNP position saturates and remains fixed with further increases of the magnetic field. This evolution suggests that the primary band structure modulation induced by the in‐plane magnetic field occurs in the vicinity of the spin transition, but before full polarization. In the hole‐doped regime (*V*_g_ ​< 10 V), the *R*–*V*_g_ curves show minimal field dependence, consistent with the weak MR response shown in Figure 2B. As highlighted in the inset of Figure 2C, the MR is negative in this regime. At *V*_g_ = 10 V (±0.5 V), a sign reversal in MR emerges, and with increasing gate voltage, the field response becomes increasingly pronounced, especially on the electron‐doped side. This asymmetric behavior with respect to the CNP underscores the interplay between spin configuration, carrier density, and field‐induced electronic reconstruction in MBT, which stands in sharp contrast to the more symmetric response observed under out‐of‐plane magnetic fields (Figure S7, Supporting Information), further emphasizing the distinct role of in‐plane field tuning.

### 2.3 Angle Dependence

**Figure 3:**
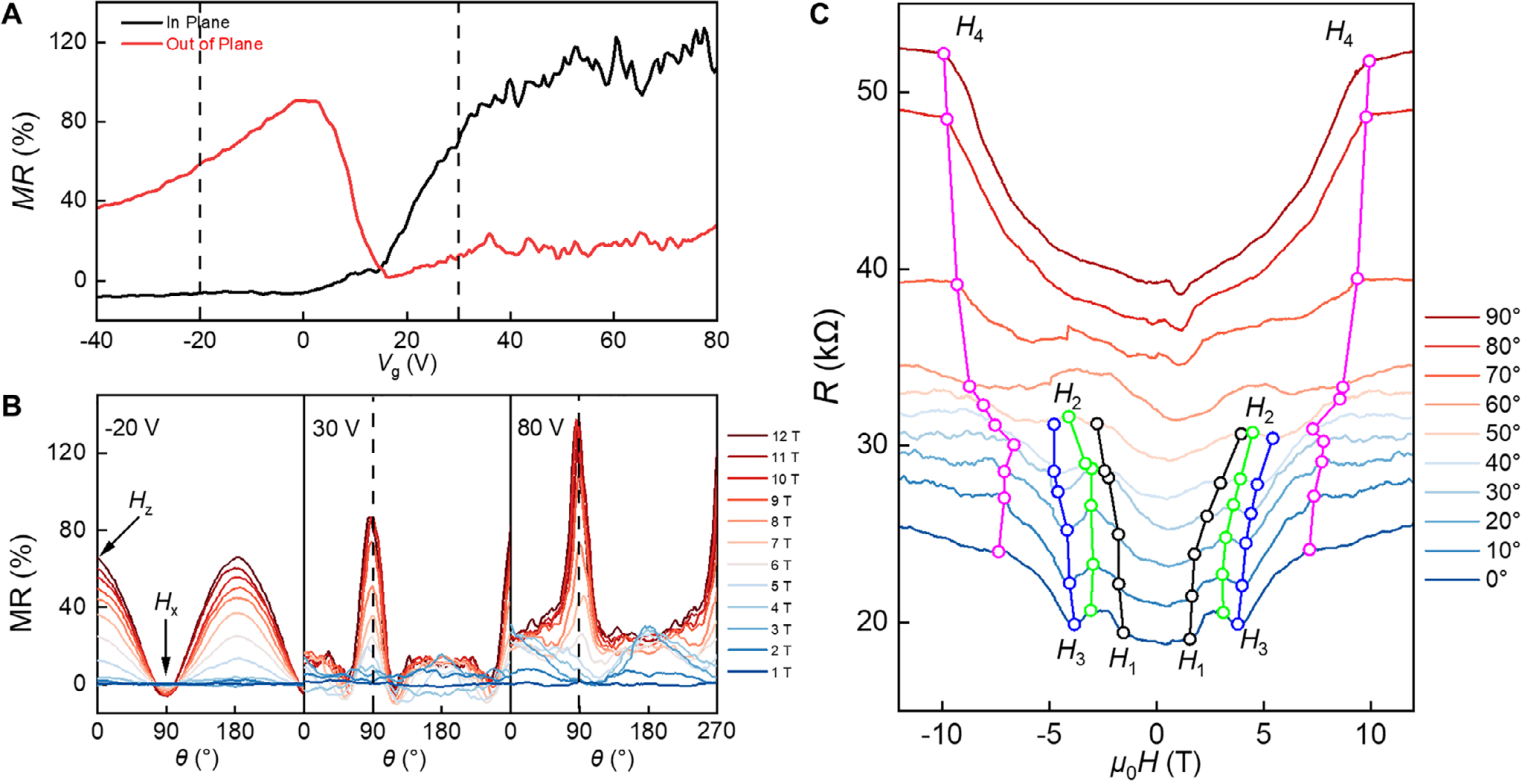
Angle‐dependent magnetotransport and characteristic field evolution in MBT. A) Comparison of MR ratio as a function of gate voltage under in‐plane (black) and out‐of‐plane (red) magnetic fields measured at 12 T. B) Angle dependence of MR ratio curves at Vg =‐−20 , 30 , and 80 V, with *θ* = 0° defined as out‐of‐plane and *θ* = 90° as in‐plane. C) MR curves measured near CNP at 1.6 K for a series of fixed magnetic field angles. Characteristic features associated with magnetic transitions ($H_{⊥}^{1}--H_{⊥}^{4}$) are marked for angles where they remain clearly resolved. Beyond ∼50°, these features become less distinguishable, and additional weak kinks appear at higher angles, whose origins remain uncertain.

To gain further insight into the evolution of magnetic ordering in MBT, we performed angle‐dependent magnetotransport measurements. **Figure** **3**A presents the MR ratio, defined as $\frac{\left[R\left(\mu_{0}H=12\;T\right)-R\left(0\right)\right]}{R(0)}$, as a function of gate voltage for magnetic fields applied either out‐of‐plane (*θ* = 0°, red curve) or in‐plane (*θ* = 90°, black curve). Under out‐of‐plane fields, the MR remains consistently positive but shows non‐monotonic behavior: it increases from 40% at large negative gate voltages to a peak near *V*_g_ = 0 V, then decreases sharply to near zero around *V*_g_ = 15 V, before gradually increasing again at higher gate voltages. In contrast, under in‐plane fields, the MR is initially negative at large negative *V*_g_​, changes sign at *V*_g_ = 12 V, and increases monotonically on the electron‐doped side, reaching 130% at *V*_g_ = 80 V. The stark contrast between the two field orientations – particularly the sign reversal seen only in the in‐plane case – underscores the anisotropic and gate‐sensitive nature of spin‐dependent scattering in MBT, likely arising from differing spin reorientation pathways and field‐induced modifications of the band structure.

To further explore the anisotropic magnetotransport behavior in MBT, we study the angle‐dependent MR at three representative gate voltages corresponding to distinct carrier regimes: *V*_g_ = −20 V (hole‐doped), 30 V (near charge neutrality), and 80 V (electron‐doped) (Figure 3B). At *V*_g_ =  −20 V, the MR ratio is largest under out‐of‐plane fields and decreases monotonically with increasing *θ*, reflecting a conventional angular dependence. In contrast, at *V*_g_ = 30 and 80 V, the MR ratio increases as the field is rotated toward the in‐plane direction, reaching a pronounced maximum at *θ* = 90°, particularly for *V*_g_ = 80 V where the MR changes by over 100%. This reversal of angular behavior and strong enhancement under in‐plane fields highlights a gate‐sensitive anisotropy that becomes prominent only in the electron‐doped regime. The data reveal that angular MR behavior in MBT is governed by both the magnetic field orientation and the underlying carrier type. See Figure S8 (Supporting Information) for additional measurements.

To further examine the angle‐dependent evolution of magnetic structure, Figure 3C presents MR curves measured near the charge neutrality point across various angles. The minor asymmetry is likely due to electrode or device asymmetry, and has negligible impact on the key transport features. For *θ* < 50°, four distinct features—labeled $H_{⊥}^{1}$ to $H_{⊥}^{4}$ (as defined in Figure 1D)—remain clearly resolved and shift to higher fields with increasing angle, consistent with the rising energy cost of spin reorientation away from the easy axis. At larger angles, these features become progressively less defined, and above *θ* > 50°, $H_{⊥}^{1}$ to $H_{⊥}^{3}$ are almost no longer distinguishable. Comparing the *θ* = 0° and 90° curves shows that the rich sequence of spin transitions ($H_{⊥}^{1}$– $H_{⊥}^{4}$) observed under out‐of‐plane fields gradually evolve into a smoother, single‐regime response under in‐plane fields. While spin reorientation still occurs under in‐plane magnetic fields, particularly between 0 T and $H_{∥}^{1}$, the intermediate transitions vanish, reflecting a continuous progression toward full FM alignment along the hard axis. These trends are consistent with observations in other A‐type antiferromagnets ,^[^ ^32^, ^33^, ^34^ ^]^ reinforcing the strong magnetic anisotropy in MBT and its highly tunable spin configuration under varying field directions. We note that at higher tilt angles, additional kinks emerge in the magnetoresistance, the origin of which remains unclear. These features may point to complexities in the spin configuration that go beyond a simple spin‐flop picture. Alternatively, device‐specific factors such as the electrode geometry could contribute to the observed asymmetry in the magnetic field response (Figure 3C). We also clarify that during device fabrication, no intentional alignment to high‐symmetry crystallographic axes was performed; the MBT flake orientation was effectively random.

### 2.4 Temperature Dependence

**Figure 4:**
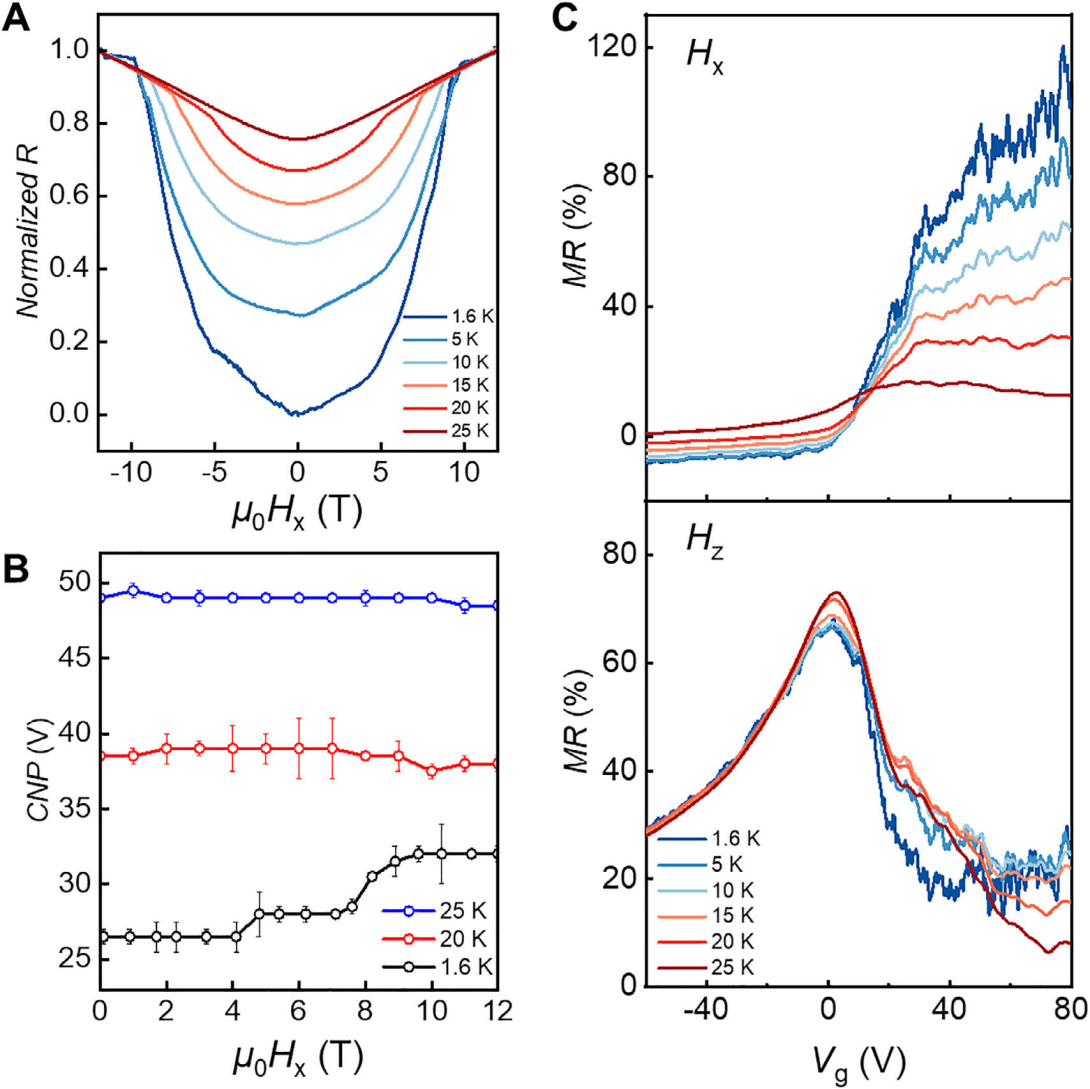
Temperature‐dependent magnetotransport of MBT. A) Normalized *R*–*B* curves under in‐plane magnetic field at different temperatures near CNP. B) CNP–*B* dependence at different temperatures under in‐plane magnetic field. C) MR–*V*_g_ curves under 12 T in‐plane field (top) and out‐of‐plane (bottom) at different temperatures.

To evaluate the temperature dependence of in‐plane magnetotransport in MBT, we study resistance as a function of in‐plane magnetic field at various fixed temperatures near CNP (**Figure** **4**A). Three key features emerge. First, the critical field required to enter the FM state decreases notably with increasing temperature: $H_{∥}^{1}$ drops from 9.7 T at 1.6 K to 5.3 T at 20 K, indicating a softening of the magnetic response. Second, at 25 K—above *T*_N_ of 22.9 K—the *R*–*μ*_0_ *H* curve exhibits different features from the spin‐reorientation behavior seen at lower temperatures. Third, in the high‐field regime beyond $H_{∥}^{1}$, the resistance continues to increase with field and becomes nearly temperature‐independent. In particular, the high‐field portions of the *R*–*μ*_0_ *H* curves converge toward the 25 K trace. To further validate this interpretation, we also measured the gate‐dependent MR at 25 K, and subtracted it from the corresponding 1.6 K gate‐dependent data (see Figure S9, Supporting Information). The resulting difference curves again exhibit an essentially flat region in the high‐field regime, suggesting that the background MR originates from a temperature‐ and gate‐insensitive contribution, possibly related to linear MR.^[^ ^11^, ^35^ ^]^ It is important to note that similar high‐field behavior is observed in other devices across different gate voltages—even when the low‐field MR sign is opposite, indicating that the rising MR background above $H_{∥}^{1}$​ is a robust feature persisting above *T*_N_​ (Figure S10, Supporting Information).

Next, we track the evolution of the CNP under in‐plane magnetic fields at various temperatures, as shown in Figure 4B. At 1.6 K, the CNP shifts substantially with increasing field, reflecting strong field‐driven modulation of the band structure. However, as the temperature increases toward *T*_N_, this effect weakens: at 20  and 25 K, the CNP position remains nearly constant with field. This trend reflects a suppression of magnetic‐field‐driven band reconstruction as long‐range magnetic order fades with increasing temperature.

Figure 4C compares gate‐dependent MR behavior under in‐plane and out‐of‐plane magnetic fields across temperatures. Under in‐plane fields, the MR at 1.6 K is initially negative for *V*_g_ < 12 V and gradually weakens with increasing temperature. This negative MR persists up to *T*_N_, consistent with a magnetic order. Above *T*_N_, the MR becomes uniformly positive across all gate voltages, although its *V*_g_ dependence maintains a similar overall shape to that observed at lower temperatures. This behavior suggests that below *T*_N_, MR is governed by magnetic scattering processes, whereas above *T*_N_, the system enters a paramagnetic regime dominated by non‐magnetic or topological contributions. In contrast, the MR–*V*_g_ response under out‐of‐plane fields exhibits minimal variation with temperature, suggesting a weaker coupling to magnetic ordering and stronger dependence on the underlying band structure.

## 3 Conclusion

In conclusion, our study demonstrates that magnetotransport in MBT is highly sensitive to in‐plane magnetic fields and gate control. Unlike the out‐of‐plane response, in‐plane fields induce a clear crossover from negative to positive MR, linked to spin reorientation and evolving scattering mechanisms. The gate‐dependent shift of the CNP under in‐plane fields reveals strong coupling between magnetic configuration and band structure. Angle‐resolved measurements uncover highly tunable magnetotransport anisotropy, while temperature‐dependent data confirm that these field‐ and gate‐induced effects persist up to *T*_N_. Together, these observations establish in‐plane magnetic fields as an effective and versatile control knob for tuning electronic and magnetic properties in MBT, paving the way for reconfigurable spintronic and topological devices based on magnetic topological insulators.^[^ ^28^, ^36^ ^]^

## 4 Experimental Section

### Heterostructure Fabrication

The h‐BN single crystals used in this study were synthesized by the temperature‐gradient method under high‐pressure and high‐temperature conditions, as previously reported in the reference.^[^ ^37^ ^]^ The MnBi_2_Te_4_ bulk crystals were grown from a Bi‐Te flux following the recipe in the reference.^[^ ^38^ ^]^ The crystals used in the devices were obtained from the same growth procedures and source batches as those characterized in the cited references. Representative optical and AFM characterizations of both crystals are shown in Figure S1 (Supporting Information). All three MnBi_2_Te_4_/h‐BN heterostructures were assembled by PDMS/PC‐assisted dry transfer method inside a nitrogen‐filled glovebox, where the O_2_ concentration was maintained below 0.5 ppm and the H_2_O level below 0.02 ppm. Thin flakes were isolated using the standard scotch‐tape exfoliation technique. A thin h‐BN flake, initially placed on a SiO_2_/Si substrate, was transferred onto an MBT flake positioned on another SiO_2_/Si substrate. The transfer polymer was removed using chloroform, followed by acetone and then IPA treatments. Electrical contact metals (Co/Au = 35/5 nm) were evaporated under a base vacuum of better than 5 × 10^−7^ mbar. The lift‐off process was carried out in an acetone/IPA solution, followed by drying with nitrogen. To maintain stability and avoid any possible degradation under ambient conditions, the encapsulated samples were stored in the glovebox under an inert atmosphere.

### Electrical Transport Measurement

Cryogenic measurements were conducted using a closed‐cycle Oxford Instruments cryomagnetic system with a base temperature of ≈ 1.6 K. A Keithley 2450 Sourcemeter was employed to apply bias through the 300 nm SiO_2_ gate dielectric. Charge transport measurements were carried out using a Stanford Research SR830 lock‐in amplifier at a low frequency of 17.777 Hz with an AC excitation current of 100 nA. Field and rotation sweeps are performed at 0.2 T min^−1^ and 0.66 ° s^−1^, respectively.

## Conflict of Interest

The authors declare no conflict of interest.

## Supporting information



Supporting Information

## Data Availability

The data that support the findings of this study are available from the corresponding author upon reasonable request.
