# Supplementary material for: Reconfigurable Magnetotransport in MnBi2Te4 via Gate and Magnetic Field Tuning
Source: Adv Mater. 2025 Sep 26;37(50):e10734. doi: 10.1002/adma.202510734 (PMC12710571; doi:10.1002/adma.202510734)
Supplement: Supplementary file 1 — Supporting Information [file ADMA-37-e10734-s001.pdf]

ADVANCED MATERIALS

Supporting Information

for *Adv. Mater.*, DOI 10.1002/adma.202510734

Reconfigurable Magnetotransport in $MnBi_2Te_4$ via Gate and Magnetic Field Tuning

*Yuang Jie, Xiaofan Cai, Yijie Lin, Kenji Watanabe, Takashi Taniguchi, Jiaqiang Yan, Dmitry Ovchinnikov and Ahmet Avsar**

ADVANCED MATERIALS

Supporting Information

Reconfigurable Magnetotransport in $MnBi_2Te_4$ via Gate and Magnetic Field Tuning

Yuang Jie[+], Xiaofan Cai[+], Yijie Lin[+], Kenji Watanabe, Takashi Taniguchi, Jiaqiang Yan, Dmitry Ovchinnikov, Ahmet Avsar[*]

Supporting Information for

Reconfigurable Magnetotransport in $MnBi_2Te_4$ via Gate and Magnetic Field Tuning

Yuang Jie+, Xiaofan Cai+, Yijie Lin+, Kenji Watanabe, Takashi Taniguchi, Jiaqiang Yan, Dmitry Ovchinnikov, Ahmet Avsar*

+ *Equal contribution*

* *Corresponding author:* aavsar@nus.edu.sg

Contents

Figure S1. Basic characterizations of flakes for Device 3.

Figure S2. Optical microscope images and gate-dependent R–$\mu_0 H$ curves of Device 1 and Device 2.

Figure S3. Optical microscope image of Device 4 (Ti/Au)

Figure S4. Gate-dependent magneto-transport of Device 4 under in-plane magnetic field at 1.6 K

Figure S5. Extraction of $H_{\parallel}^{1}$ from $dR/d\mu_0 H$ colormap

Figure S6. Additional two-terminal magneto-transport measurements of Device 4 under in-plane magnetic fields at 1.6 K

Figure S7. Gate-dependent magneto-transport under out-of-plane magnetic field at 1.6 K

Figure S8. Angle-dependent magneto-transport at other representative gate voltages

Figure S9. R– $\mu_0 H$ curves at 1.6 K and 25 K with different gate voltages.

Figure S10. Normalized R– $\mu_0 H$ curves of Device 1 and Device 2 under in-plane magnetic field at different temperatures

Figure S11. **R_{xx} and R_{xy} – $\mu_0 H$ of Device 2 under out-of-plane magnetic field at different gate voltages.**

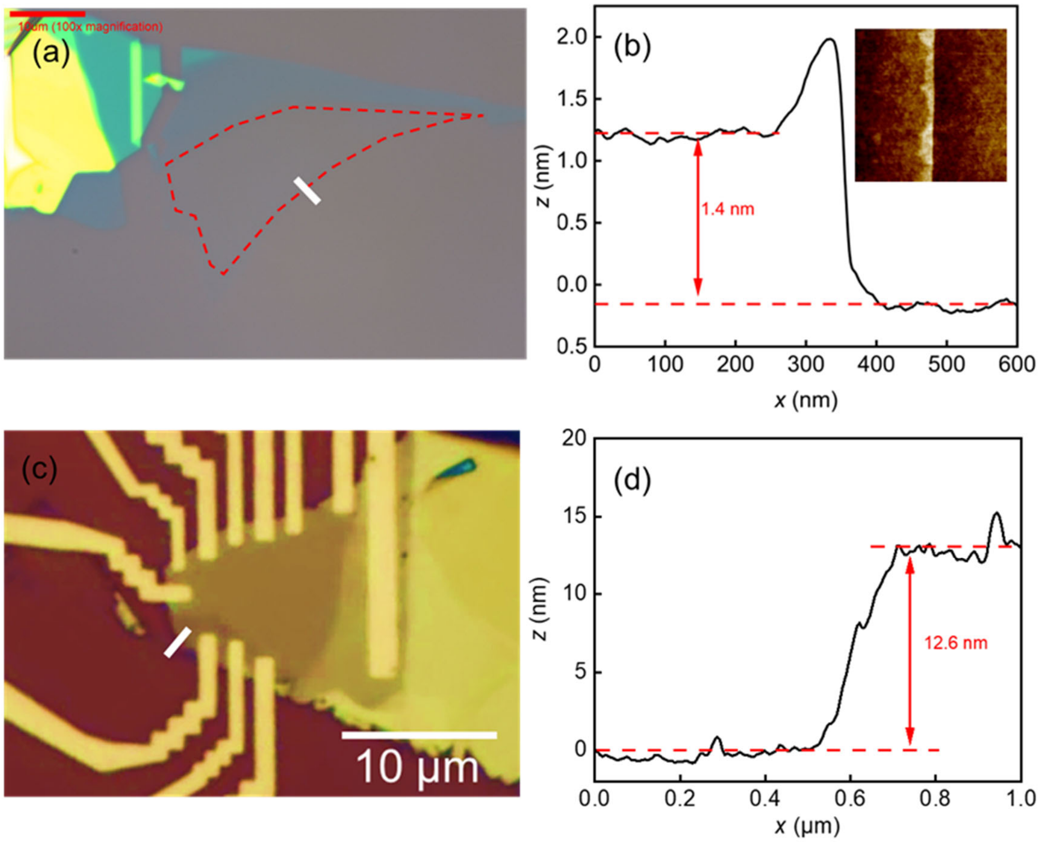

Figure S1. (a): Optical image of h-BN. The red dashed line indicates the thin h-BN. White line represents the direction measured by AFM. (b): The thickness of the h-BN is 1.4 nm. Inset is the accurate area. (c): Optical image of MBT flake used for Device 3. (d): Device 3 with a thickness of 12.6 nm measured by atomic force microscopy. The red dashed line indicates the height profile along the white line in (c).

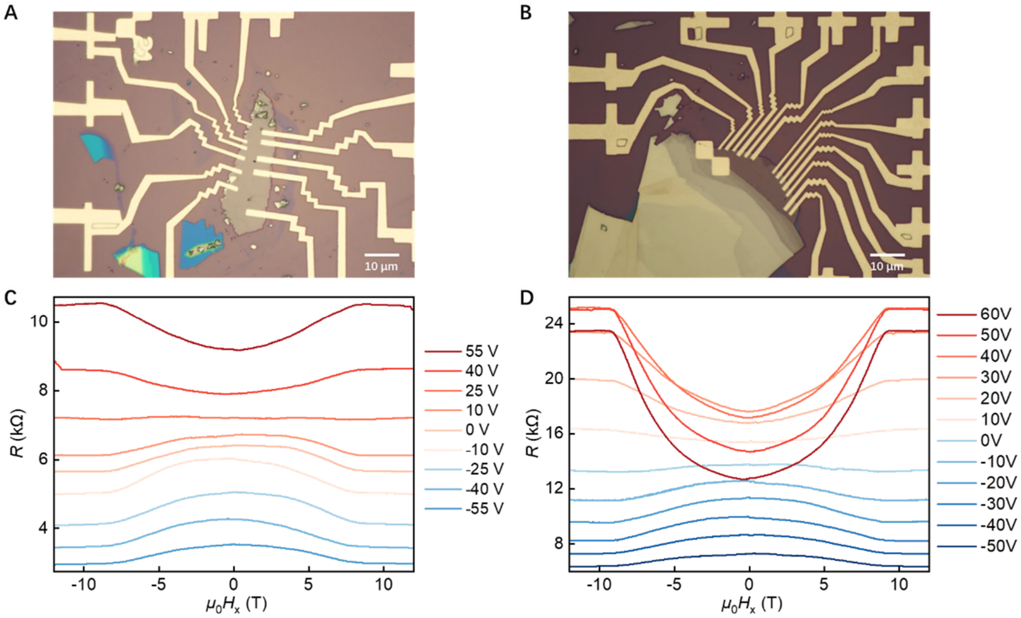

Figure S2. Optical microscope images of A) Device 1 and B) Device 2. Gate-dependent R– $\mu_0 H$ curves of C) Device 1 and D) Device 2 under an in-plane magnetic field at 1.6 K, exhibit a similar negative-to-positive magnetoresistance crossover with gate voltage as observed in Device 3.

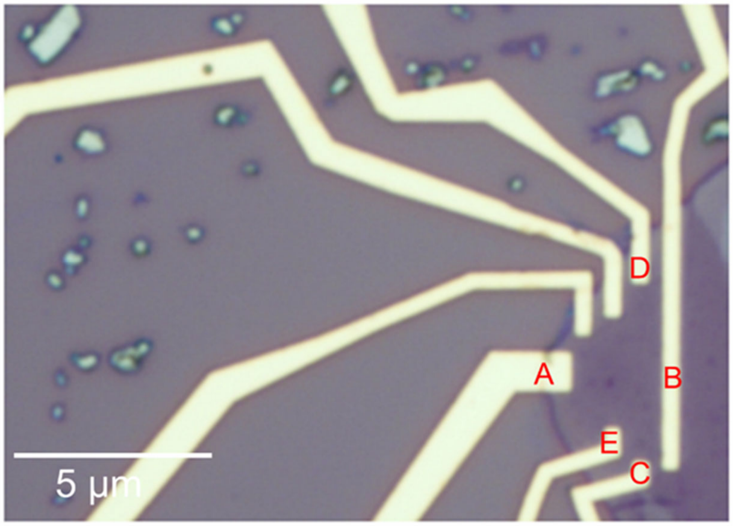

Figure S3: Optical microscope image of Device 4, contacted with Ti/Au metals. Electrode A and B are source and drain, while C and D are used for hall measurement. Electrode A, B, C, E are designed for four-terminal measurements.

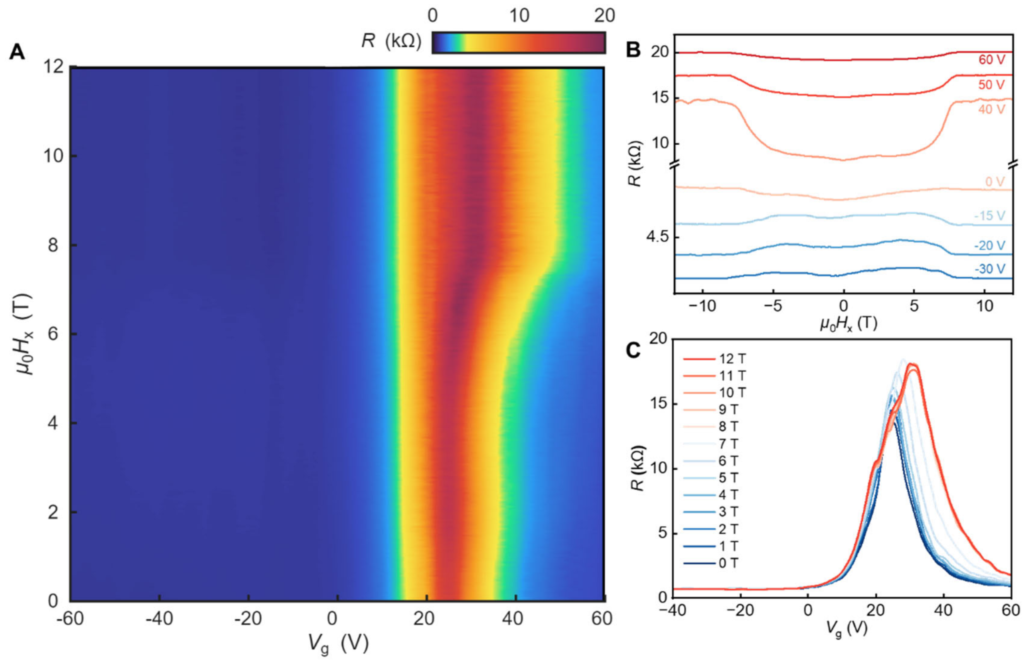

Figure S4: Gate-dependent magnetotransport of Device 4 with Ti/Au contacts under in-plane magnetic field at 1.6 K. A) Colormap of resistance as a function of in-plane magnetic field and gate voltage B) R–μ_0H curves under in-plane magnetic field at different gate voltages, vertically offset for clarity. C) Four-terminal resistance as a function of magnetic field at different in-plane magnetic fields.

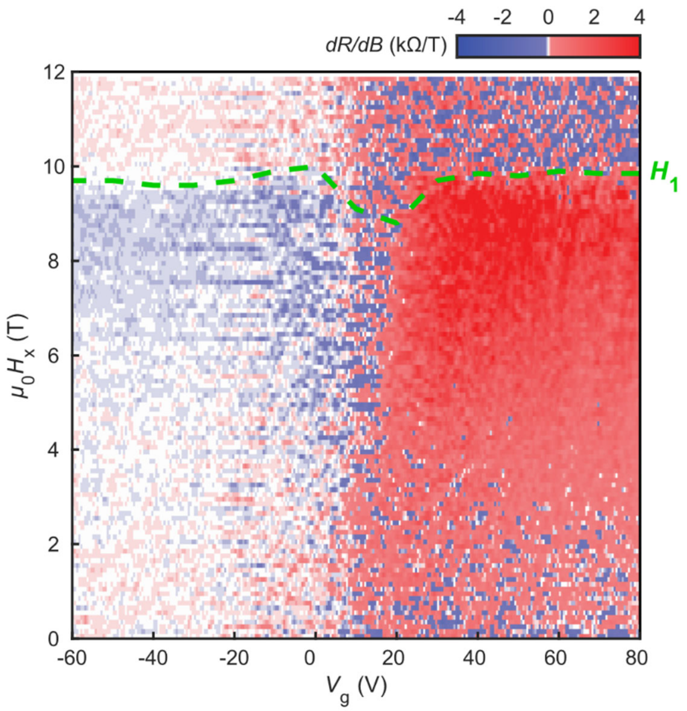

Figure S5. $dR/d(\mu_0 H)$ colormap as a function of in-plane magnetic field and gate voltage at 1.6 K, the green dashed line indicates the position of characteristic field H_1.

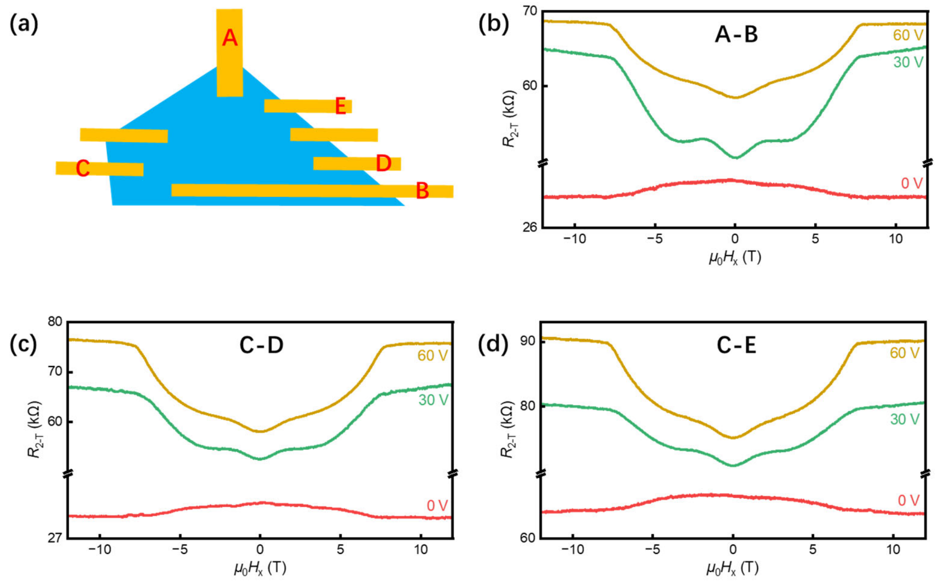

Figure S6: a) Schematic of Device 4 for two-terminal measurement. The blue region represents the $MnBi_2Te_4$ flake, while the yellow bars denote the Ti/Au electrodes. A–E label the specific electrodes. b-d) R–B curves under in-plane magnetic field at different gate voltages, vertically offset for clarity. Two-terminal resistances were measured using electrodes A-B, C-D and C-E, respectively.

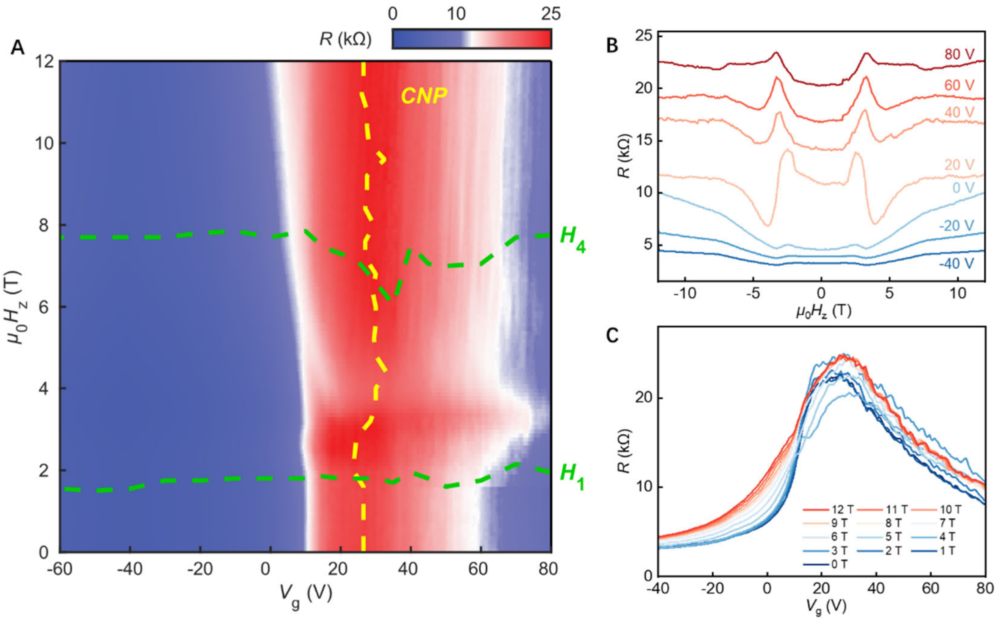

Figure S7. Gate-dependent magneto-transport of MBT under out-of-plane magnetic field at 1.6 K. A) Colormap of resistance as a function of out-of-plane magnetic field and gate voltage. The green dashed line are the characteristic fields H_1 and H_4 and the yellow dashed line represents evolution of the CNP. B) R– $\mu_0 H$ curves under out-of-plane magnetic field at various gate voltages, vertically offset for clarity. C) R–V_g curves at different out-of-plane magnetic fields.

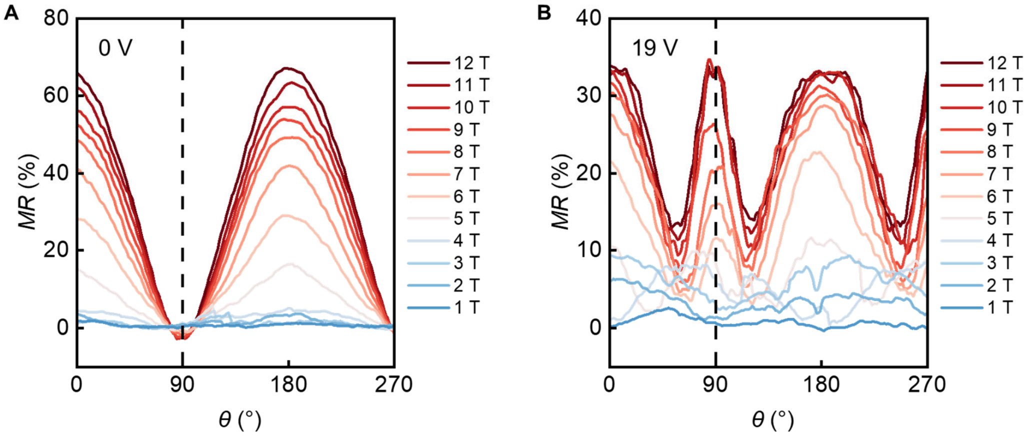

Figure S8. Angle dependence curves with increasing magnetic fields at V_g = A) 0 V and B) 19 V, with θ = 0° defined as out-of-plane and θ = 90° as in-plane.

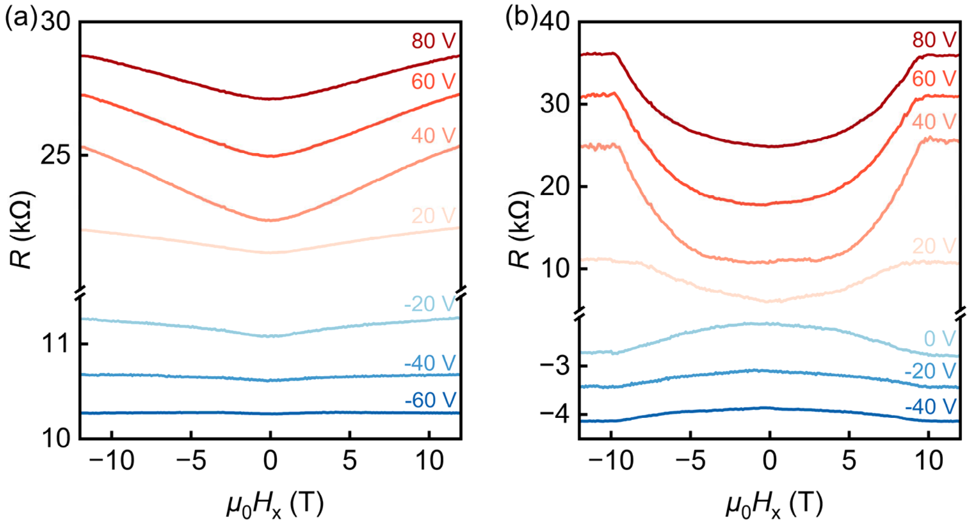

Figure S9: (a): R– μ_0H curves under in-plane magnetic field at 25 K with different gate voltages. (b): R– μ_0H curves under in-plane magnetic field at 1.6 K with different gate voltages after subtracting data at 25 K. Both panels use vertical offsets to separate the curves.

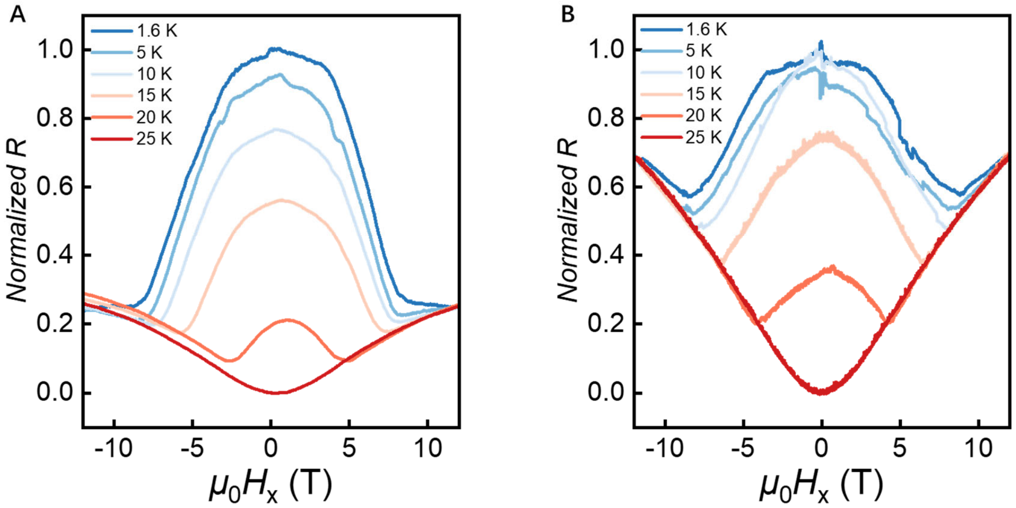

Figure S10. Normalized R– μ_0H curves of A) Device 1 and B) Device 2 at different temperatures under in-plane magnetic field at $V_g = 0$ V.

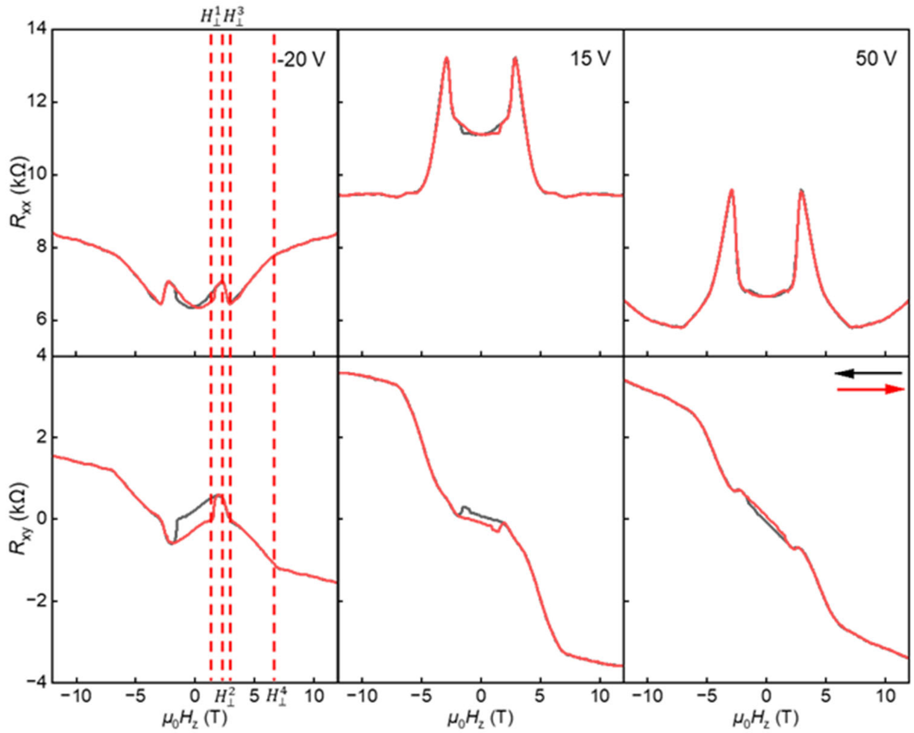

Figure S11: $R_{xx} - \mu_0 H$ and $R_{xy} - \mu_0 H$ of Device 2 under out-of-plane magnetic field at different gate voltages. Black and red arrows indicate the sweeping direction of magnetic field. Red dashed lines indicate four characteristic fields $H_\perp^1$, $H_\perp^2$, $H_\perp^3$ and $H_\perp^4$.
